# LKB1 inhibits proliferation, metastasis and angiogenesis of thyroid cancer by upregulating SIK1

**DOI:** 10.7150/jca.72021

**Published:** 2022-07-04

**Authors:** Bo Kou, Xin-Di Wang, Xiao-Peng Sun, Qin Qi, Ming Yang, Yan-Ning Yun, Jin-Song Zhou, Wei Liu

**Affiliations:** 1 Department of Otorhinolaryngology-Head&Neck Surgery, The First Affiliated Hospital of Xi'an Jiaotong University, Xi'an, Shaanxi 710061, China.; 2 Department of Clinical Medicine, Medical School of Xian Jiaotong University, Xi'an, Shaanxi 710061, China.; 3 Department of Otorhinolaryngology-Head&Neck Surgery, The Second Affiliated Hospital of Xi'an Medical University, Xi'an, Shaanxi, 710038, China.; 4 Department of Cardiology, Xianyang Hospital of Yan'an University, Xianyang, Shaanxi Province, 712000, China.; 5 Department of Otorhinolaryngology-Head&Neck Surgery, The First Affiliated Hospital of Xi'an Medical University, Xi'an, Shaanxi, 710001, China.; 6 Department of Human Anatomy, Histology and Embryology, School of Basic Medical Sciences, Xi'an Jiaotong University Health Science Center, Xi'an, Shaanxi 710061, China.; 7 Key Laboratory of Environment and Genes Related to Diseases, Xi'an Jiaotong University, Ministry of Education of China, Xi'an, Shaanxi 710061, China.; 8 Department of Vascular Surgery, The First Affiliated Hospital of Xi'an Jiaotong University, Xi'an, Shaanxi 710061, China.

**Keywords:** LKB1, thyroid cancer, metastasis, EMT, angiogenesis, SIK1

## Abstract

**Purpose:** Liver kinase B1 (LKB1), also known as serine/threonine kinase 11, was considered as a tumor suppressor, which exhibited anti-cancer activity in a variety of cancers. However, the effect of LKB1 in thyroid cancer remains unclear.

**Methods:** In the study, MTT assay, colony formation assay, flow cytometry, western blot analysis, wound healing assay, transwell assays, quantitative real-time PCR, HUVEC migration assay, ELISA assay, tube formation assay and nude mice xenograft were used to investigate the anti-cancer capacity of LKB1 in thyroid cancer *in vitro* and *in vivo*.

**Results:** In the present study, we found that the expression of LKB1 was lower in thyroid cancer tissues and cell lines, compared with the adjacent normal tissue and thyroid epithelial cell. After construction of stable clone cells with ectopic LKB1 overexpression, the findings revealed that LKB1 overexpression exerted anti-proliferative and pro-apoptotic property in thyroid cancer TPC-1 and BCPAP cells. In addition, LKB1 overexpression could inhibit migration and invasion, downregulate MMP2 and MMP9 expressions, and reverse EMT in thyroid cancer cells. Furthermore, overexpression of LKB1 attenuated HUVEC recruitment, decreased the expression of VEGFA and inhibited the formation of new vessels in thyroid cancer cells. To validate the underlying mechanism of LKB1 in thyroid cancer, the results showed that LKB1 could positively regulate SIK1 in thyroid cancer TPC-1 and BCPAP cells. Additionally, the SIK1 inhibitor HG-9-91-01 could partially abrogate the anti-proliferative and anti-metastatic effect of LKB1, and reverse MET (mesenchymal-to-epithelial transition) mediated by LKB1 overexpression. Ultimately, the results *in vivo* revealed that LKB1 overexpression exhibited a strong inhibitory effect of tumorigenicity and presented anti-angiogenic characteristic in nude mice xenograft model.

**Conclusion:** the results demonstrated that LKB1 could inhibit proliferation, metastasis phenotype and angiogenesis, and reverse EMT in thyroid cancer *in vitro* and *vivo* via the upregulation of SIK1, suggesting that LKB1 could be considered as a potential therapeutic target for the treatment of thyroid cancer.

## Introduction

Thyroid cancer is one of the most common endocrine malignancies worldwide. In United States, there is an estimated 44,280 new cases and 2,200 deaths in 2021 1. In developing counties, the mortality and morbidity of thyroid cancer have a remarkable increase, which gained a great attention. Among all types of thyroid cancer, the papillary thyroid cancer (PTC) is the most prevalent form 2. In spite of the advances in diagnostic tools and treatment for PTC, there is still a great challenge for the treatment of PTC patients with recurrence and metastasis. Therefore, it's of great urgency to verify the possible mechanisms of thyroid cancer recurrence and metastasis.

Liver kinase B1 (LKB1), also known as serine/threonine kinase 11, was first identified in Peutz-Jeghers syndrome 3. It was recognized as a tumor suppressor, and took part in a variety of biological behaviors, such as cell metastasis, polarity, self-renewal and energy metabolism, and LKB1 exhibited anti-cancer activity in various cancers 4-6. Studies showed that LKB1 could modulate lung cancer differentiation and metastasis 7. In addition, LKB1 was reported to inhibit breast cancer proliferation 8-9. Furthermore, the activation of LKB1 may suppress proliferation of gastric cancer via the inhibition of the nuclear translocation of YAP and β-catenin 10. But, the effect of LKB1 in human thyroid cancer has not been elucidated yet.

In the present study, we aimed to verify the role of LKB1 in thyroid cancer proliferation, migration, invasion, epithelial-mesenchymal transition and angiogenesis, as well as the possible underlying mechanism of LKB1 in modulation of these biological behavior.

## Methods and materials

### Cell culture and reagents

Human thyroid cancer cell lines TPC-1, BCPAP, thyroid cell line Nthy-ori 3-1 and human umbilical vein endothelial cell line (HUVEC) were purchased from the American Type Culture Collection (Manassas, VA, USA). The cell lines were maintained in 1640 medium, supplemented with 10% fetal bovine serum (Gibco, Grand Island, NY, USA) and seeded at 37 °C in a humidified incubator with 5% CO_2_ humidity.

Antibodies against phosphorylated-LKB1 (p-LKB1 Ser428), LKB1, Caspase 3, poly ADP-ribose polymerase (PARP), Matrix Metallopeptidase 2 (MMP2), MMP9, E-Cadherin (E-Ca), N-Cadherin (N-Ca), Vimentin and β-actin were obtained from Cell Signaling Technology, Inc (Beverly, MA, USA). Antibodies against phosphorylated-SIK1 (Salt inducible kinase 1, Thr182), SIK1 and VEGFA (Vascular endothelial growth factor-A) was purchased from Abcam, Inc (Cambridge, Britain). The ELISA kit was obtained from RayBiotech Inc (Norcross, GA, USA). 3-(4,5-dimethylthiazol-2-yl)-2,5-diphenyltetrazolium bromide (MTT) was purchased from Sigma Chemical Co. (St. Louis, MO, USA).

### Clinical specimens

Thyroid cancer patients' tissues and adjacent normal tissues (n=5) were obtained from Department of pathology, The First Affiliated Hospital of Xi'an Jiaotong University. All clinical specimens were approved by the Ethics Committee of Hospital, and informed consent were obtained from all the patients (n=5) enrolled in the study. Then the isolated tissues were prepared for the subsequent western blotting assay.

### Western blotting

Cells and tissues were washed with PBS buffer after certain treatment, and the proteins were extracted using a lysis buffer. After centrifugation and denaturation, equal amounts of the extracts (about 30-60 µg) were loaded into SDS (Sodium Dodecyl Sulfate)-polyacrylamide gel electrophoresis (10 % or 15 %) and electrotransferred onto polyvinylidene fluoride membranes (Millipore, Bedford, MA, USA) for 2 h. Membranes were then incubated with primary antibodies against p-LKB1, LKB1, Caspase 3, PARP, MMP2, MMP9, E-Ca, N-Ca, Vimentin, p-SIK1, SIK1, VEGFA and β-actin overnight at 4 °C, respectively. Subsequently, the membranes were washed with TBS buffer and incubated with horseradish peroxidase (HRP)-conjugated IgG antibody at room temperature (25 °C) for 1 h. Ultimately, the protein bands were visualized using an enhanced chemiluminescence kit (Bio-Rad, CA, USA) and exposed to X-ray film.

### Plasmid transfection

The lentivirus overexpressing LKB1 (OE-LKB1) and vector were constructed by GeneCopoeia (Guangzhou, China). The stable clone cells were used for the subsequent experiments after lentivirus transfection with additional 8 μg/ml polybrene.

### Quantitative real-time PCR assay

The total RNA of cells was extracted using TRIzol reagent (Invitrogen, Carlsbad, CA, USA) and reversely transcribed to complementary DNA (cDNA) using a PrimerScript RT reagent Kit (Takara, Dalian, China). Then the quantitative real-time PCR (Polymerase Chain Reaction) was conducted using FAST SYBR Green Master Mix. The gene-specific primers for PCR amplification were as follows: forward 5'-TGTCGGTGGGTATGGACAC-3'and reverse 5'-CCTTGCCGTAAGAGCCTTCC-3' for LKB1 (135 bp); forward 5'-CCCACTGCGGTTTTCTCGAAT-3' and reverse 5'-CAAAGGGGTATCCATCGCCAT-3'for MMP2 (89 bp); forward 5'-AGACCTGGGCAGATTCCAAAC-3' and reverse 5'-CGGCAAGTCTTCCGAGTAGT-3' for MMP9 (94 bp); forward 5'-CGAGAGCTACACGTTCACGG-3' and reverse 5'-GGGTGTCGAGGGAAAAATAGG-3' for E-Cadherin (119 bp); forward 5'-TCAGGCGTCTGTAGAGGCTT-3' and reverse 5'-ATGCACATCCTTCGATAAGACTG-3' for N-Cadherin (94 bp); forward 5'-GACGCCATCAACACCGAGTT-3' and reverse 5'-CTTTGTCGTTGGTTAGCTGGT-3' for Vimentin (238 bp); forward 5'-CATGTACGTTGCTATCCAGGC-3' and reverse 5'-CTCCTTAATGTCACGCACGAT-3' for β-actin (250 bp). The n-fold change in mRNAs expression was calculated with the 2^-ΔΔCt^ method. The experiments were performed in triplicate.

### MTT assay

MTT assay was used to detect the viability of the thyroid cancer cells. Briefly, cells with a concentration of 20,000 per well were seeded into 96-well plates. After incubation with certain treatment for different times (0, 24, 48, 72 h), 20 μl MTT dye solution (5.0 mg ml^-1^) was added to each well with 180 μl medium. Four hours later, cells were lysed with dimethyl sulfoxide (DMSO) to dissolve the formazan crystals. Then the absorbance was measured at 490 nm wavelength by microplate reader (Bio-Rad, Hercules, CA, USA). The experiments were performed in triplicate.

### Colony formation assay

After resuspension, thyroid cancer cells with a concentration of 1000 cells per well were seeded into 6-well plates and incubated with 1640 medium for two weeks. After washing with PBS buffer for three times, the cells were fixed with 4% paraformaldehyde for 15 mins and stained with 0.1% crystal violet for another 10 mins. The clonogenicity was visualized and counted at 100×magnification. The experiments were performed in triplicate.

### Apoptosis detection

Flow cytometry was performed to detect the cell apoptosis. Cells under certain treatments for 24 h were collected and washed with PBS buffer. Then cells were stained with fluorescein isothiocyanate (FITC)-conjugated annexin V and propidium iodide (PI) using Annexin V-FITC detection kit (KeyGen, Nanjing, Jiangsu, China) according to the manufacturer's instructions. Then the apoptotic cells were analyzed with BD FACScan flow cytometer (BD Biosciences, San Diego, CA, USA). The experiments were performed in triplicate.

### Wound healing assay

Cells were collected and seeded into six-well plates. After the cell density reached to the whole plate, the scratch wounds were made by dragging a 200-µl pipette tip across the monolayer. Subsequently, the scratches were observed in a serum-free medium at different times (0, 24 h), and images (100 ×) were then taken under an inverted microscope (Olympus IX50, Tokyo, Japan) to explore the migratory ability. The experiments were performed in triplicate.

### Cell migration assay

Thyroid cancer TPC-1 and BCPAP cells were chosen for this assay under certain conditions. 2 × 10^4^ cells in 200 μl serum-free medium were seeded onto the upper chambers, and 800 μl of 10 % fetal calf serum-containing medium was added in the lower chamber in 96-well plate. After incubation for 24 h, the cells in the upper chamber was removed with a cotton swab. Subsequently, the migrated cells in the lower surface of the filter were fixed with 4 % paraformaldehyde, and stained with 0.1% crystal violet (Beyotime, Shanghai, China). Cells were then counted and photographed in five random fields under an inverted microscope (Olympus IX50, Tokyo, Japan) at 100 × magnification.

### Matrigel invasion assay

Millicell chamber (Millipore, Billerica, MA, USA) was used in matrigel invasion assay to detect the cell invasive ability. The transwell chambers were pre-coated with 50 μl Matrigel (Matrigel: serum-free medium 1:5). After 4 h, thyroid cancer cells with a concentration of 6 × 10^4^ cells in 200 μl serum-free medium were seeded onto the upper chambers, and 800 μl medium with 10% fetal calf serum was added to the lower chamber. The subsequent protocols were similar to that in the cell migration assay.

### Conditional medium collection and ELISA assay

Thyroid cancer TPC-1 and BCPAP cells were seeded and adhered into 6-well plate. After washed with serum-free medium (SFM), cells were exposed to certain treatment with SFM. The supernatant was then collected and centrifuged to obtain the conditional medium (CM).

With the collected conditional medium (CM), the concentration of VEGFA in CM was measured by the ELISA kit following the manufacturer's protocol. The experiments were performed in triplicate.

### HUVEC migration assay

The assay was similar to that in the cell migration assay. 3 × 10^4^ HUVEC cells in 200 μl serum-free medium were seeded into the upper chambers, and 800 μl conditional medium (CM) from TPC-1 or BCPAP sublines, or co-cultured system (HUVEC cells with TPC-1 or BCPAP sublines) were added in the lower chamber. After incubation for certain times, the cells in the upper chamber were removed with a cotton swab. Followed by the cell fixation and staining, cells were counted and photographed under an inverted microscope at 100 × magnification.

### Tube formation assay

After trypsinization, HUVEC cells were re-suspended with the conditional medium (CM). Subsequently, cells were seeded into 24-well plates, which were pre-coated with Matrigel in a total of 50 μl each well (Matrigel: serum-free medium=1:5). After incubation for four hours, the tube was observed and counted under an inverted microscope at 100 × magnification.

### Xenograft tumor model

Four-week-old male BALB/C nude mice were purchased from and maintained in the Animal Care and Use Committee of Xi'an Jiaotong University, Xi'an, China. All the animal experiments were approved by the Ethics Committee of Animal care and Use of Xi'an Jiaotong University. Twelve mice were randomly and equally separated into two groups, and then subcutaneously injected into right flank with 100 μL serum-free medium containing 5×10^6^ stable clone cells (TPC-1 vector or OE-LKB1). The tumor diameter and mice weight were measured every 3 days, and the tumor volume was calculated as 1/2 × (length) × (width)^2^. Finally, the nude mice were sacrificed after 30 days and xenografts were isolated for the subsequent western blotting assay.

### Bioinformatics and statistical analysis

The expressions of LKB1 and SIK1 in thyroid cancer and normal tissues, and the correlations between LKB1/SIK1 and E-Ca were analyzed with database GEPIA (http://gepia.cancer-pku.cn/). Data were represented as mean ± standard deviation*.* All statistical analyses were conducted by GraphPad Prism (vesion 6.0) software. Statistical differences in different groups were compared by Student's *t*-test (two-sided) or one-way analysis of variance (ANOVA). *P <* 0.05 was considered as statistical significance.

## Results

### The expressions of LKB1 in thyroid cancer tissues and cell lines, and the construction of stable clone cells

**Figure 1:**
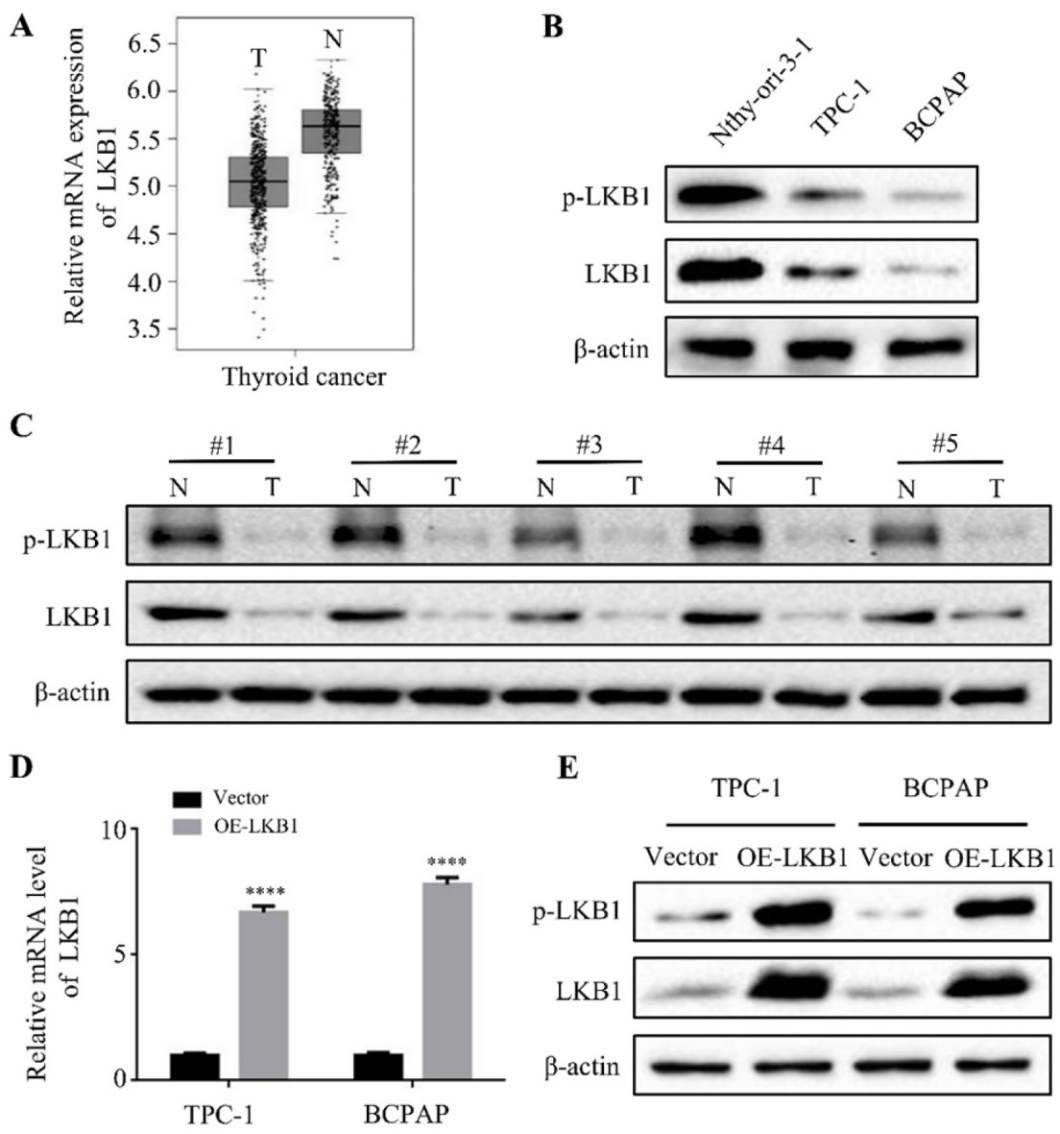
** The expressions of LKB1 in thyroid cancer tissues and cell lines, and the construction of stable clone cells.** (A) The mRNA expression of LKB1 in thyroid cancer obtained from database GEPIA. (B) Western blot was used to detect the protein levels of phosphorylated-LKB1 and total-LKB1 in (B) thyroid epithelial Nthy-ori-3-1 cells, thyroid cancer TPC-1 and BCPAP cell lines, and (C) 5 cases of human thyroid cancer tissues and adjacent normal tissues. N: adjacent normal tissue; T: thyroid cancer tissue. (D) Quantitative real-time PCR and (E) western blot was conducted to detect the mRNA and protein level of LKB1 in stable clone cells with LKB1 overexpression or vector, respectively. Quantification from three independent experiments was shown with error bars representing standard deviation (SD). ****, *P*<0.0001.

Firstly, to explore the expressions of LKB1 in thyroid cancer, the bioinformatics analysis was used. The results of database GEPIA showed that the mRNA expression of LKB1 was higher in adjacent normal tissues than that in thyroid cancer tissues (Figure **1A**). Consistent with the bioinformatics data, the protein levels of phosphorylated-LKB1 (p-LKB1) and total LKB1 significantly increased in human thyroid epithelial Nthy-ori 3-1 cell, compared with that in thyroid cancer TPC-1 and BCPAP cell lines (Figure **1B**). In addition, LKB1 was also upregulated in the adjacent normal tissues compared with its corresponding thyroid cancer tissues, as detected by the western blot assay (Figure **1C**). Based on this criterion, the stable clone cell lines with overexpression of LKB1 were constructed. The results revealed a remarkable increase of LKB1 mRNA and protein level in TPC-1 and BCPAP cells with LKB1 overexpression, as evidenced by quantitative real-time PCR and western blot, respectively (Figure **1D** and **1E**).

### LKB1 overexpression inhibited thyroid cancer cell proliferation *in vitro*

**Figure 2:**
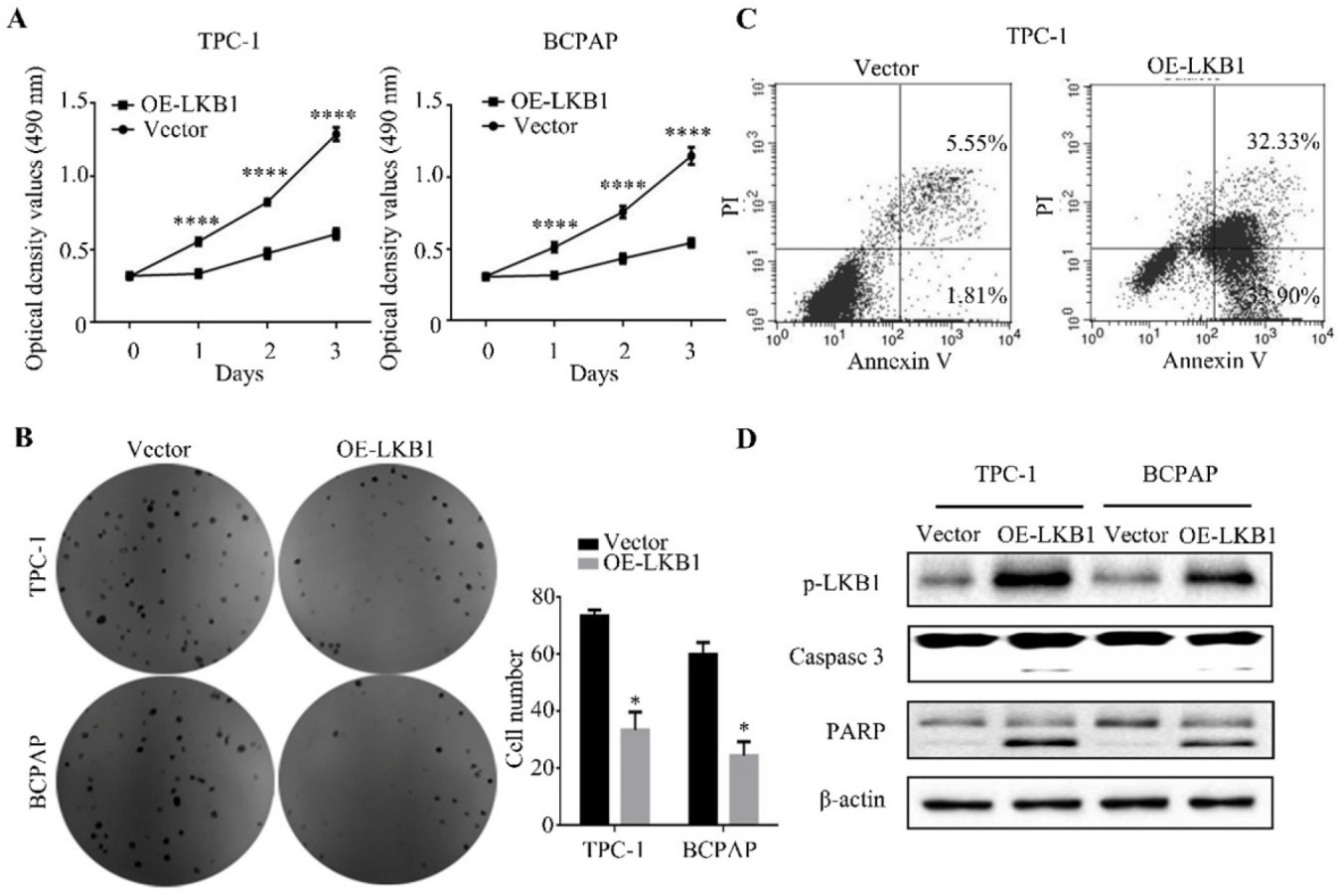
** LKB1 overexpression inhibited thyroid cancer cell proliferation *in vitro.*** (A) MTT assay and (B) colony formation assay were performed to measure the cell viability of TPC-1 or BCPAP cells with LKB1 overexpression or vector. Quantification was shown with error bars representing standard deviation (SD). *, *P*<0.05 and ****, *P*<0.0001. (C) The apoptosis induction in thyroid cancer TPC-1 cells with treatments as indicated was analyzed by flow cytometry. (D) The pro-apoptotic proteins caspase 3 and PARP in stable clones with LKB1 overexpression or vector were detected by western blot. β-actin was used as a loading control.

The MTT assay showed that LKB1 overexpression dramatically suppressed the proliferation of thyroid cancer TPC-1 and BCPAP cells in a time-dependent manner (Figure **2A**). In accordance with the above results, the findings of colony formation assay demonstrated that overexpressing LKB1 could inhibit cell viability in these two thyroid cancer cells (Figure **2B**). Meanwhile, the flow cytometry analysis revealed that LKB1 overexpression could induce apoptosis in thyroid cancer cells (Figure **2C**). And apoptosis-related proteins, including caspase 3 and PARP, were found to be induced in TPC-1 and BCPAP cells overexpressing LKB1 (Figure **2D**). These data suggested that LKB1 overexpression inhibited thyroid cancer cell proliferation capacity.

### LKB1 overexpression inhibited metastatic phenotype of thyroid cancer cell *in vitro*

**Figure 3:**
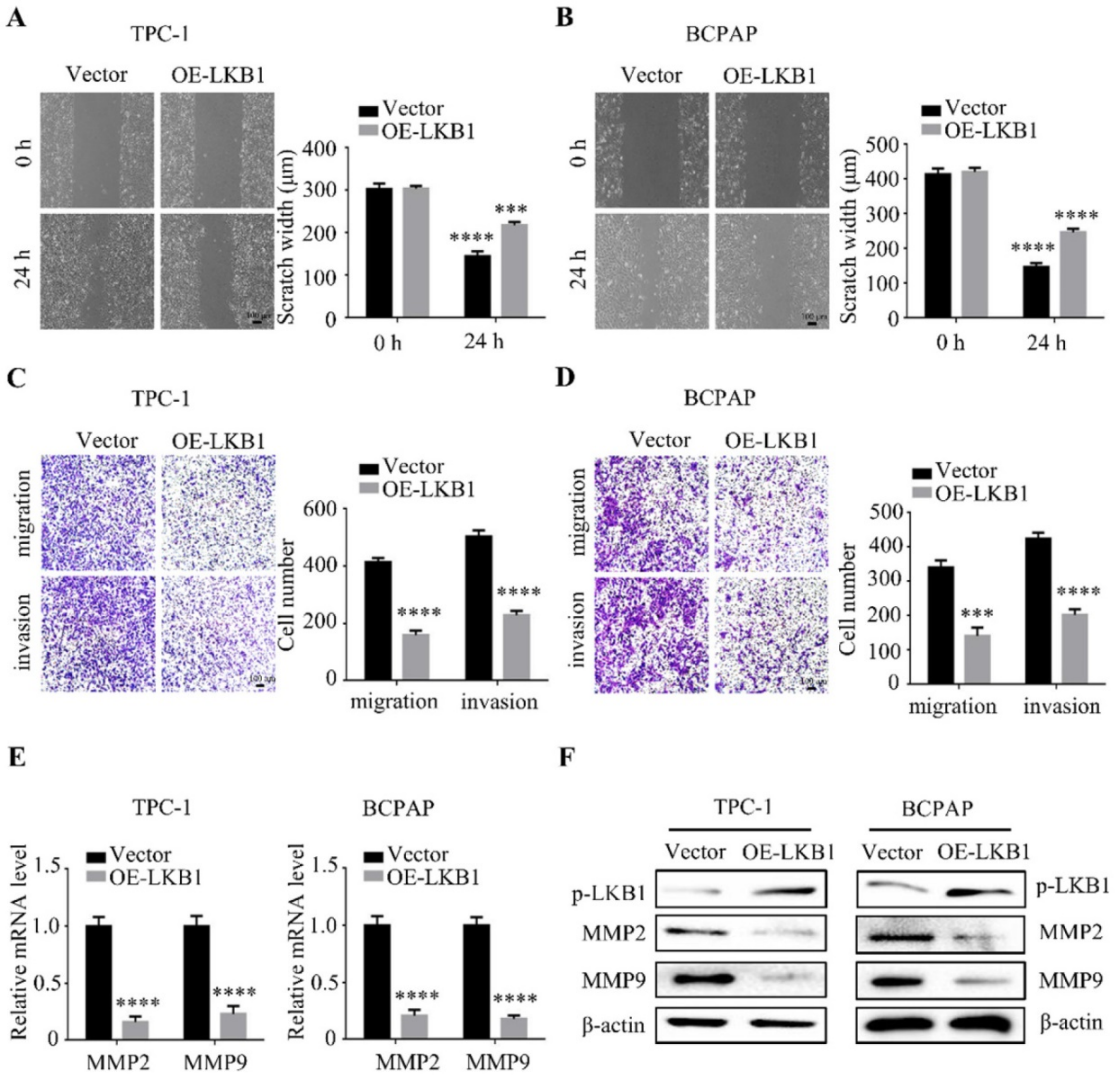
** LKB1 overexpression inhibited metastatic phenotype of thyroid cancer cell *in vitro.*** Representative images of wound healing in (A) TPC-1 and (B) BCPAP cells with vector or LKB1 overexpression. Quantification from three independent experiments was shown with error bars representing standard deviation (SD). The migration and invasion ability in (C) TPC-1 and (D) BCPAP cells with LKB1 overexpression was assessed by transwell migration assay and matrigel invasion assay. Quantification analysis was shown on the right. The mRNA (E) and protein (F) levels of MMP2 and MMP9 were detected by quantitative real-time PCR and western blot in thyroid cancer cells with vector or LKB1 overexpression, respectively. β-actin was used as a loading control. ***, *P*<0.001 and ****, *P*<0.0001.

To further explore the function of LKB1 in thyroid cancer cell metastasis, wound healing assay and transwell assays were performed simultaneously after LKB1 overexpression. The data showed that overexpressing LKB1 could impede the scratch compared with that in vector group (Figure** 3A** and** 3B**). Similar with the above results, LKB1 overexpression could also suppress migration and invasion ability of thyroid cancer TPC-1 and BCPAP cells (Figure **3C** and **3D**). Matrix metalloproteinases, a member of proteases, have been reported to participate in the cancer metastasis and progression. In the present study, we found that the mRNA and protein levels of MMP2 and MMP9 were remarkably decreased in TPC-1 and BCPAP cells after LKB1 overexpression (Figure **3E** and **3F**). All the data indicated that LKB1 played an inhibitory role in metastatic phenotype of thyroid cancer cells.

### LKB1 overexpression reversed epithelial-mesenchymal transition of thyroid cancer cells

**Figure 4:**
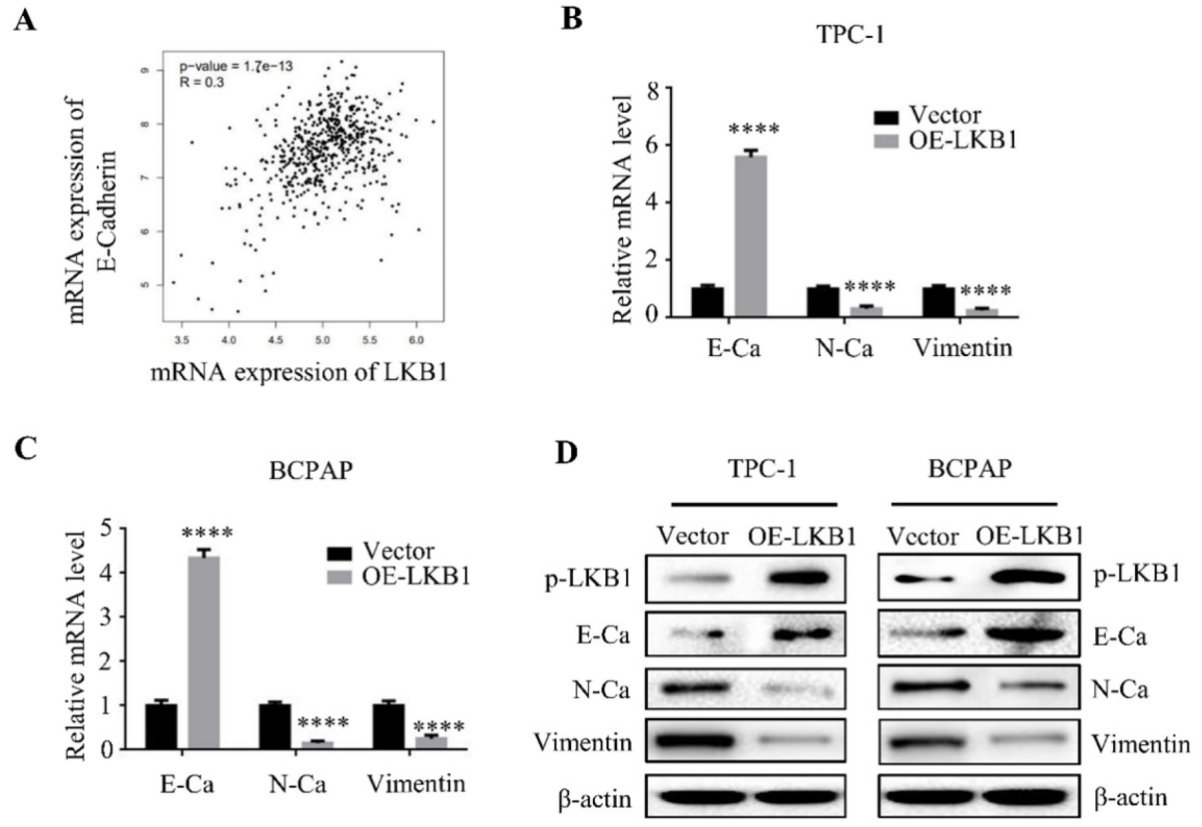
** LKB1 overexpression reversed epithelial-mesenchymal transition of thyroid cancer cells.** (A) The correlation between LKB1 and E-Cadherin expression obtained from database GEPIA. The mRNA (B-C) and protein (D) levels of E-Ca, N-Ca and vimentin were assessed by quantitative real-time PCR and western blot in thyroid cancer cells with vector or LKB1 overexpression, respectively. β-actin was used as a loading control. ****, *P*<0.0001.

Epithelial-mesenchymal transition (EMT) is a hallmark of cancer metastasis. Studies stated that LKB1 is involved with the regulation of EMT in various cancers 11-12. As expected, the database analysis demonstrated that the mRNA expression of LKB1 was positively correlated with the expression of E-Ca (Figure **4A**). To validate the role of LKB1 in thyroid cancer EMT, quantitative real-time PCR and western blot assay were conducted to verify the hypothesis. The findings showed that overexpression of LKB1 exhibited a remarkable increase of mRNA and protein levels of E-Ca, whereas a decrease of N-Ca and vimentin expressions in thyroid cancer TPC-1 and BCPAP cells (Figure **4B** and **4C**). The similar results were also observed in the western blot assay (Figure **4D**). The results suggested that LKB1 could inhibit EMT of thyroid cancer cells.

### LKB1 overexpression blocked angiogenesis of thyroid cancer cells

**Figure 5:**
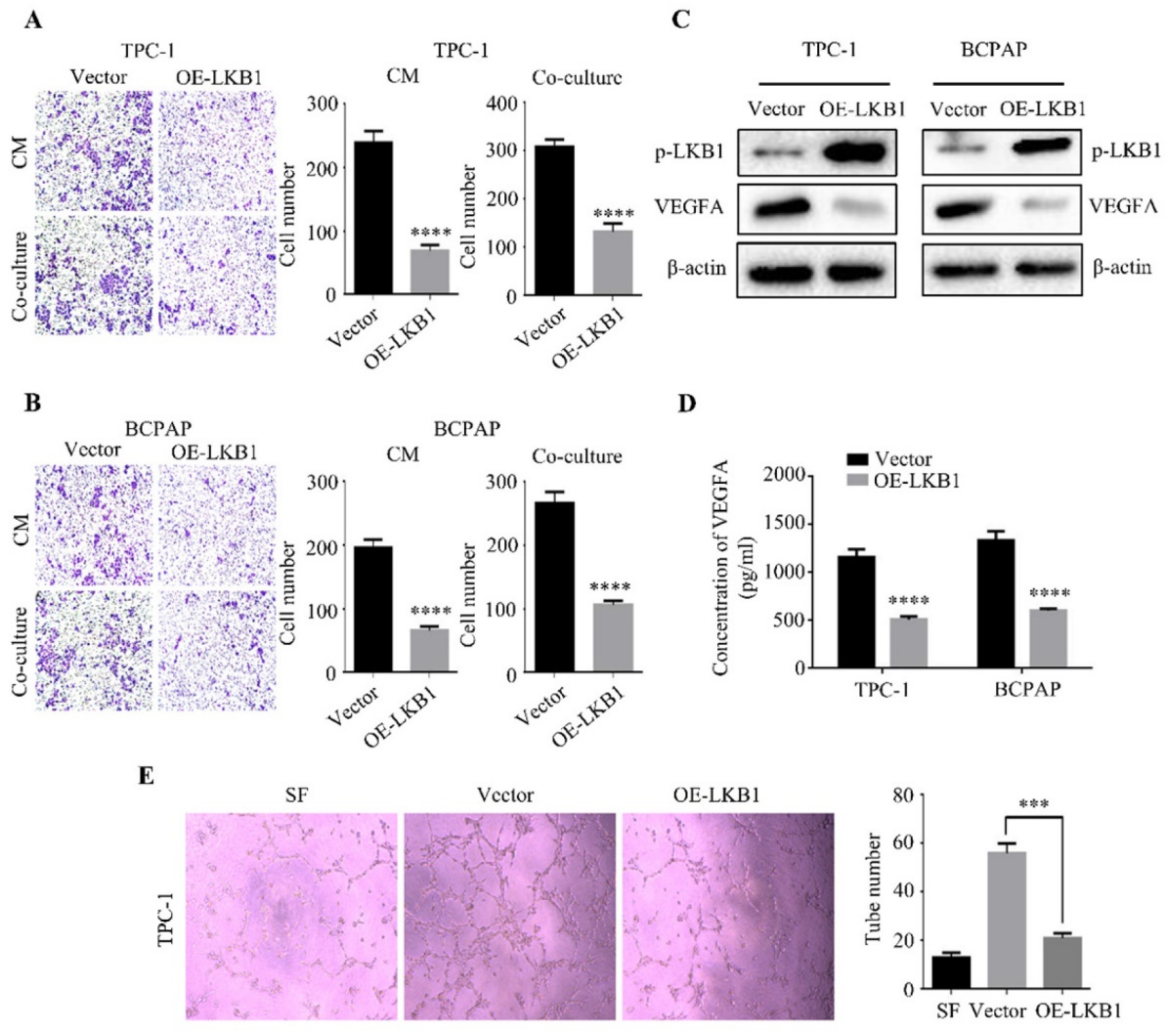
** LKB1 overexpression blocked angiogenesis of thyroid cancer cells.** Representative images of HUVEC recruitments in the conditional mediums (CMs) from (A) TPC-1 or (B) BCPAP sublines, or co-cultured mediums. Quantification analysis was shown on the right. (C) The protein level of VEGFA in TPC-1 and BCPAP cells with vector or LKB1 overexpression was shown by western blotting. β-actin was used as a loading control. (D) The concentration of VEGFA in conditional mediums (CMs) with treatments as indicated was detected by ELISA assay. (E) Tube formation assay was used to evaluate the formation of new vessels. The representative images and quantification analysis of tube number of HUVECs in conditional mediums (CMs) with treatments as indicated were shown (magnification, ×100). ***, *P*<0.001 and ****, *P*<0.0001.

Besides EMT, angiogenesis was also closely associated with cancer progression 13. So we put an insight into the correlation between LKB1 and angiogenesis, and HUVEC migration assay was performed to evaluate the HUVEC recruitment induced by thyroid cancer cells. HUVECs were seeded into the upper chamber, while we added the conditional medium (CM) from TPC-1 or BCPAP sublines or co-cultured the stable clone cells in the lower chamber. The results showed that LKB1 overexpression in TPC-1 and BCPAP cells could decrease the recruitment of HUVECs (Figure **5A** and **5B**). To further confirm the role of LKB1 in thyroid cancer angiogenesis, we detected a crucial regulatory gene of tumor angiogenesis, VEGFA. Indeed, the protein level of VEGFA was downregulated in TPC-1 or BCPAP cells with LKB1 overexpression (Figure **5C**). Furthermore, ELISA data indicated that overexpressing LKB1 could decrease the secretion of VEGFA in these two thyroid cancer cells (Figure **5D**). These results suggested that LKB1 regulated VEGFA expressions to participate in thyroid cancer angiogenesis. Then, tube formation assay was conducted to detect the effect of LKB1 on the formation of new vessels. Consistent with the above results, the data demonstrated that conditional medium from TPC-1 LKB1-overexpressed sublines could suppress the formation of tube-like structure in matrigel (Figure **5E**). Taken together, the above findings revealed that LKB1 overexpression could inhibit thyroid cancer angiogenesis *in vitro*.

### LKB1 overexpression inhibited tumor growth, metastasis and EMT by upregulation of SIK1 in thyroid cancer cells

**Figure 6:**
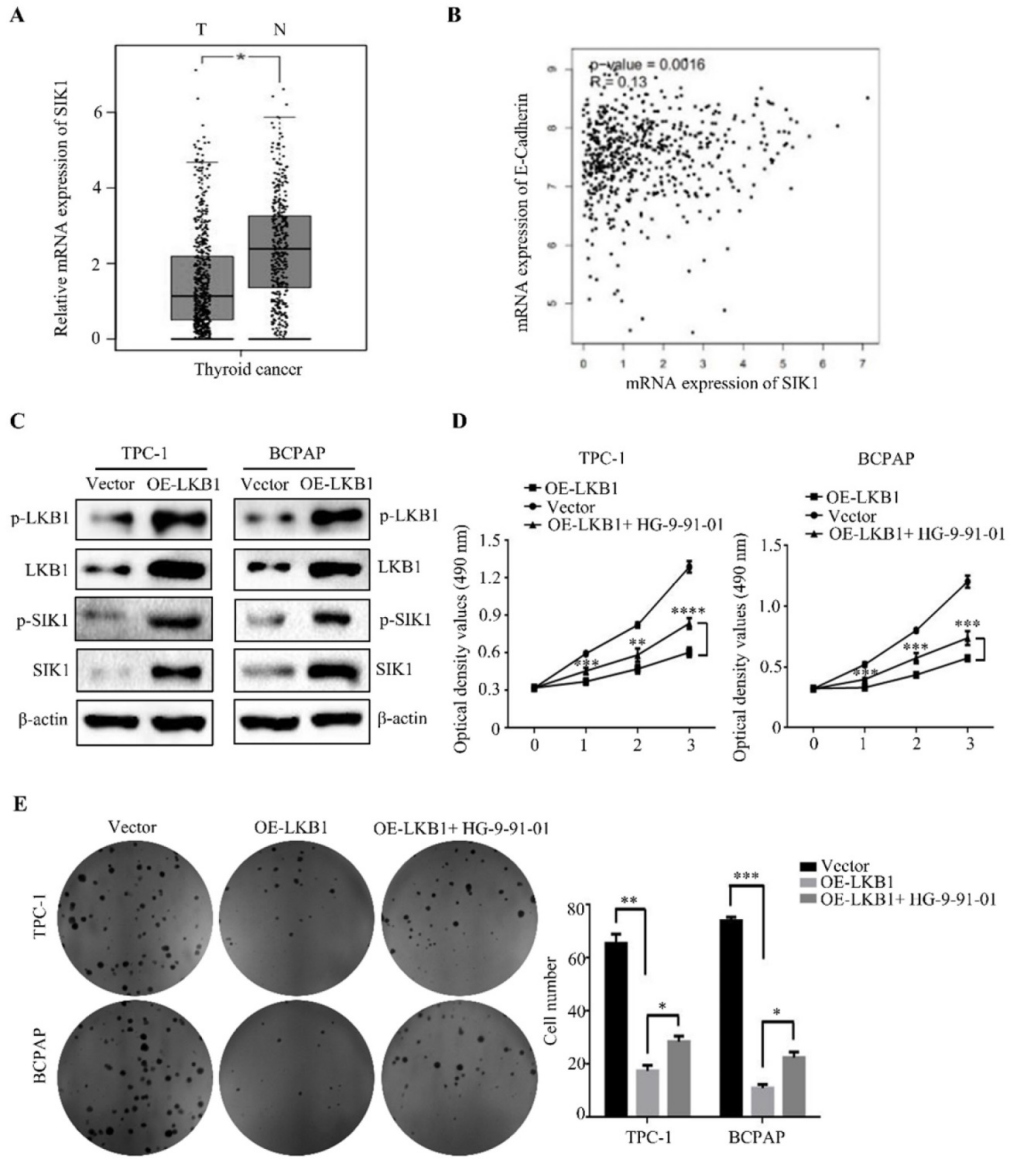
** LKB1 overexpression inhibited tumor growth by upregulating SIK1 in thyroid cancer cells.** (A) The mRNA expression of SIK1 in thyroid cancer, and (B) the correlation between SIK1 and E-Cadherin expression obtained from database GEPIA. (C) The protein levels of phosphorylated-LKB1, total-LKB1, phosphorylated-SIK1 and total-SIK1 in TPC-1 and BCPAP cells with vector or LKB1 overexpression were shown by western blotting. β-actin was used as a loading control. (D) MTT assay and (E) colony formation assay were performed to evaluate the viability of TPC-1 and BCPAP cells with vector or LKB1 overexpression after treatment with SIK1 inhibitor HG-9-91-01. Quantification was shown with error bars representing standard deviation (SD). *, *P*<0.05, **, *P*<0.01, ***, *P*<0.001, and ****, *P*<0.0001.

**Figure 7:**
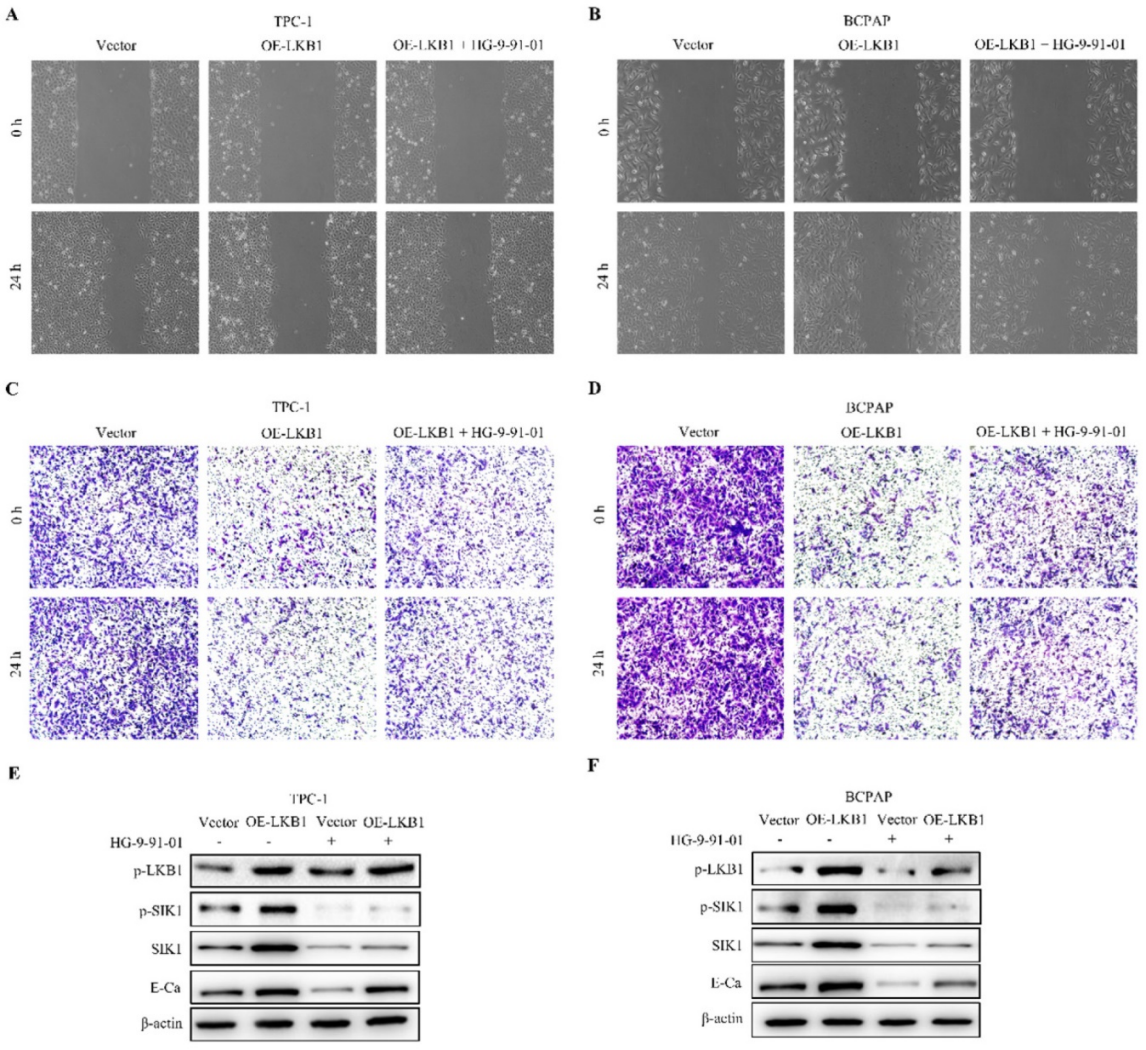
** LKB1 overexpression inhibited metastasis and EMT by upregulation of SIK1 in thyroid cancer cells.** (A-B) Wound healing assay and (C-D) transwell assays were conducted in TPC-1 and BCPAP cells with vector or LKB1 overexpression, treated as described above. (E-F) The protein levels of phosphorylated-LKB1, phosphorylated-SIK1, SIK1 and E-ca were detected by western blot in TPC-1 and BCPAP cells after treatment as described above. β-actin was used as a loading control.

To elucidate the underlying mechanism of anti-cancer property of LKB1 in thyroid cancer cells, we detected the downstream regulators of LKB1 in thyroid cancer. We found that the expression of SIK1 was significantly lower in thyroid cancer tissues than that in normal tissues (Figure **6A**). Moreover, the mRNA level of SIK1 was positively correlated with E-Ca level (Figure **6B**), and overexpression of LKB1 could upregulate p-SIK1 and SIK1 protein levels in thyroid cancer TPC-1 and BCPAP cells (Figure **6C**). To further explore whether SIK1 was involved with antagonism of LKB1 in thyroid cancer cells or not, HG-9-91-01, a specific SIK1 inhibitor, was introduced. The results uncovered that HG-9-91-01 partially abrogated the proliferation inhibition mediated by LKB1 overexpression in thyroid cancer TPC-1 and BCPAP cells, as evidenced by MTT assay and colony formation assay (Figure **6D** and** 6E**). For the metastatic phenotype, the inhibition of SIK1 by HG-9-91-01 reversed the anti-migratory and anti-invasive effect of LKB1 overexpression to some extent, as confirmed by wound healing assay, transwell migration and matrigel invasion assay (Figure **7A-7D**). Consistently, the results of western blotting clearly stated that the elevation of E-Ca by LKB1 overexpression could also be reversed in TPC-1 and BCPAP cells after HG-9-01-01 treatment (Figure **7E** and **7F**). All the data collectively supported that the anti-oncogenic capacity of LKB1 in thyroid cancer was mediated by the positive regulation of SIK1.

### LKB1 overexpression inhibited tumorigenicity and angiogenesis of thyroid cancer *in vivo*

**Figure 8:**
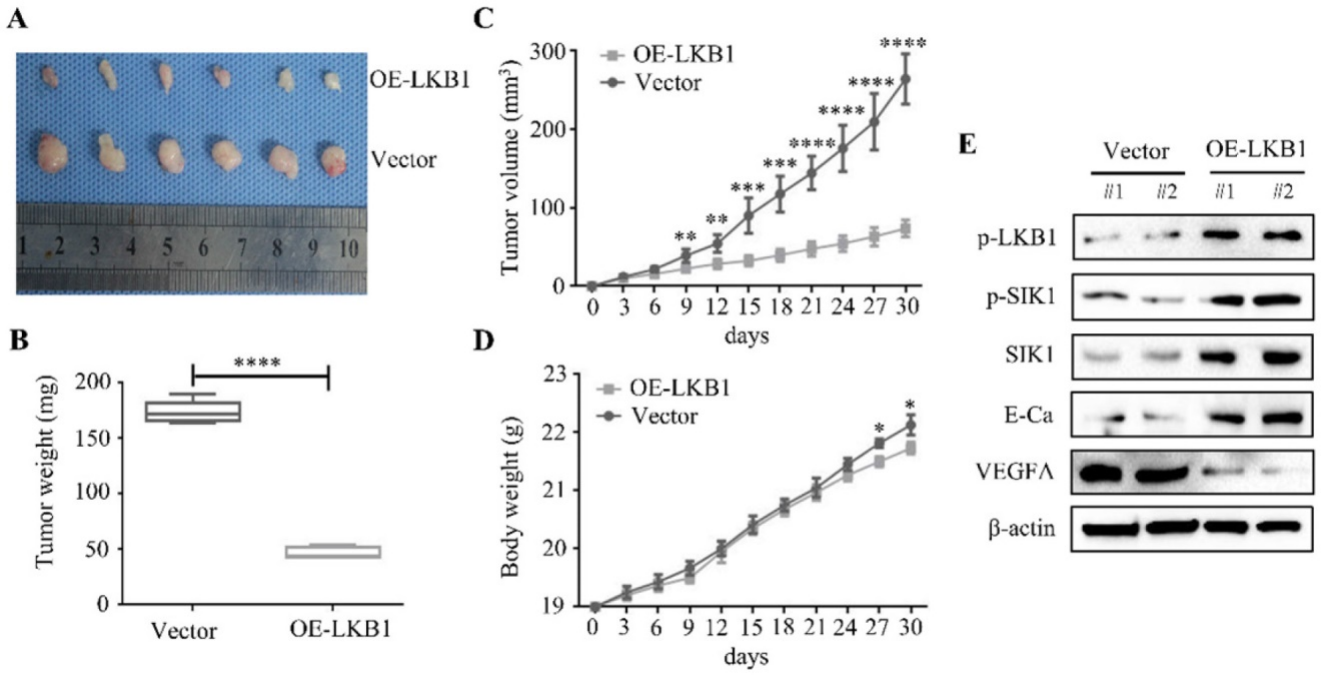
** LKB1 overexpression inhibited tumorigenicity and angiogenesis of thyroid cancer *in vivo*.** (A) Representative images and quantification analysis of xenografts in TPC-1 cells with vector or LKB1 overexpression were shown in 30 days. 5×10^6^ TPC-1 cells were subcutaneously injected into the right flank of nude mice. (B) The quantification analysis of tumor mass in vector and LKB1 overexpression groups were shown as mean ± SD (****P < 0.0001). (C) Tumor volumes and (D) body weight of nude mice were detected every 3 days. The statistical results were presented as mean ± SD of six mice (*P < 0.05, **P < 0.01, ***P < 0.001, ****P < 0.0001). (E) The protein levels of phosphorylated-LKB1, phosphorylated-SIK1, SIK1, E-Ca and VEGFA were detected by Western blot. β-actin was used as a loading control. The proteins were collected from the dissected tumor tissues of vector and LKB1 overexpression groups.

A nude mice xenograft was established to explore the therapeutic effect of LKB1 *in vivo*. The representative image of xenograft was shown in Figure **8A**, and the findings revealed that the LKB1 overexpression group presented a prominent decrease in the tumor mass and volume, compared with the vector group (Figure **8B** and** 8C**). Whereas there was few significant difference in body weight in these two groups (Figure **8D**). Then western blotting results from isolated tumor tissues showed that the protein levels of p-SIK1, SIK1 and E-Ca were upregulated, while VEGFA expression was downregulated in LKB1 overexpression group (Figure **8E**). Taken together, these findings indicated that LKB1 overexpression attenuated the tumorigenicity of thyroid cancer *in vivo*.

## Discussion

So far, there are only a few studies reporting the effect of LKB1 in thyroid cancer. One study showed that (V600E) BRAF inhibition could induce the cytoprotective autophagy via LKB1-AMPK signaling in thyroid cancer cells 14. In the present study, the protein levels of p-LKB1 and LKB1 were remarkably lower in thyroid cancer tissues and cell lines, compared with the adjacent normal tissue and thyroid epithelial cell, respectively. After construction of stable clone cells with ectopic LKB1 overexpression, the findings uncovered that LKB1 overexpression exhibited anti-proliferative property in thyroid cancer TPC-1 and BCPAP cells, which was consistent with the previous studies 15-16. Meanwhile, overexpression of LKB1 could induce apoptosis and upregulate the pro-apoptotic protein levels in thyroid cancer cells, as confirmed by flow cytometry and western blot analysis.

Metastasis is a complicated cellular process, including the degradation of the extracellular matrix (ECM), epithelial-mesenchymal transition, tumor angiogenesis 17. Studies reported that LKB1 could regulate invasion and metastasis in colorectal cancer 18. And studies showed that the loss of LKB1 accelerated breast cancer metastasis and invasion 19. In the present study, the results demonstrated that overexpression of LKB1 could inhibit migration and invasion of thyroid cancer TPC-1 and BCPAP cells, and downregulate the expressions of MMP2 and MMP9. In addition, the mRNA and protein levels of E-Ca were dramatically increased, while the expressions of N-Ca and vimentin were significantly decreased in TPC-1 and BCPAP cells with LKB1 overexpression. For the angiogenesis, LKB1 was also found to suppress lung cancer angiogenesis by Shh signaling pathway 20. Depletion of LKB1 could accelerate the angiogenesis via VEGF 21. In this study, the data revealed that LKB1 overexpression attenuated the HUVEC recruitments and decreased the expression of VEGFA in thyroid cancer cells, as evidenced by HUVEC migration assay, western blot and ELISA assay. And the following tube formation assay indicated that LKB1 could inhibit the formation of new vessels. All the results supported that LKB1 overexpression could suppress the metastasis and angiogenesis of thyroid cancer cells.

To explore the underlying mechanism, LKB1-SIK1 signaling pathway has been reported to participate in a variety of biological behaviors 22-23. Studies revealed that activation of LKB1-SIK1 signaling blocked the TGF-β-mediated EMT and induced apoptosis in ovarian carcinoma 24. And studies showed that LKB1-SIK1 signaling could attenuate EMT and radioresistance of non-small cell lung cancer cells 25. Consistent with the previous studies, LKB1 was found to positively regulate SIK1 in thyroid cancer TPC-1 and BCPAP cells. Furthermore, the SIK1 inhibitor HG-9-91-01 could partially abrogate anti-proliferative and anti-metastatic effect of LKB1, and reverse MET mediated by LKB1 overexpression. These results suggested that LKB1-SIK1 signaling was involved with thyroid cancer progression. Additionally, a nude mice xenograft was established and the results *in vivo* revealed that LKB1 overexpression exhibited a strong inhibitory effect of tumorigenicity and presented anti-angiogenic characteristic in thyroid cancer tissue.

Taken together, the results of the present study revealed, for the first time, that LKB1 could inhibit proliferation, metastasis phenotype and angiogenesis, and reverse EMT in thyroid cancer* in vitro* and *vivo* via the upregulation of SIK1. And the findings strongly suggested that LKB1 could be considered as a potential therapeutic target for the treatment of thyroid cancer.

## References

[1] Siegel RL, Miller KD, Fuchs HE, Jemal A (2021). Cancer statistics, 2021. CA Cancer J Clin.

[2] Abdullah MI, Junit SM, Ng KL, Jayapalan JJ, Karikalan B, Hashim OH (2019). Papillary thyroid cancer: genetic alterations and molecular biomarker investigations. Int J Med Sci.

[3] Tacheci I, Kopacova M, Bures J (2021). Peutz-Jeghers syndrome. Curr Opin Gastroenterol.

[4] Caja L, Dadras MS, Mezheyeuski A, Rodrigues-Junior DM, Liu S, Webb AT (2022). The protein kinase LKB1 promotes self-renewal and blocks invasiveness in glioblastoma. J Cell Physiol.

[5] Shackelford DB, Shaw RJ (2009). The LKB1-AMPK pathway: metabolism and growth control in tumour suppression. Nat Rev Cancer.

[6] Gao Y, Yan Y, Tripathi S, Pentinmikko N, Amaral A, Paivinen P (2020). LKB1 represses ATOH1 via PDK4 and energy metabolism and regulates intestinal stem cell fate. Gastroenterology.

[7] Ji H, Ramsey MR, Hayes DN, Fan C, McNamara K, Kozlowski P (2007). LKB1 modulates lung cancer differentiation and metastasis. Nature.

[8] Zhuang Z, Wang K, Cheng X, Qu X, Jiang B, Li Z (2013). LKB1 inhibits breast cancer partially through repressing the hedgehog signaling pathway. PLos One.

[9] Sengupta S, Nagalingam A, Muniraj N, Bonner MY, Mistriotis P, Afthinos A (2017). Activation of tumor suppressor LKB1 by honokiol abrogates cancer stem-like phenotype in breast cancer via inhibition of oncogenic Stat3. Oncogene.

[10] Ma LG, Bian SB, Cui JX, Xi HQ, Zhang KC, Qin HZ (2016). LKB1 inhibits the proliferation of gastric cancer cells by suppressing the nuclear translocation of Yap and beta-catenin. Int J Mol Med.

[11] Hu M, Zhao T, Liu J, Zou Z, Xu Q, Gong P (2019). Decreased expression of LKB1 is associated with epithelial-mesenchymal transition and led to an unfavorable prognosis in gastric cancer. Hum Pathol.

[12] Qin Z, Xie R, Zhang W, Wan M, Zhang L, Wang H (2020). Liver kinase B1 correlates with prognosis and epithelial-mesenchymal transition of resectable early stage non-small cell lung cancer. Transl Cancer Res.

[13] Ribatti D (2017). Epithelial-mesenchymal transition in morphogenesis, cancer progression and angiogenesis. Exp Cell Res.

[14] Jimenez-Mora E, Gallego B, Diaz-Gago S, Lasa M, Baquero P, Chiloeches A (2021). (V600E) BRAF inhibition induces cytoprotective autophagy through AMPK in thyroid cancer cells. Int J Mol Sci.

[15] Zhang K, Wang J, Wang J, Luh F, Liu X, Yang L (2019). LKB1 deficiency promotes proliferation and invasion of glioblastoma through activation of mTOR and focal adhesion kinase signaling pathways. Am J Cancer Res.

[16] Wang YQ, Dai WM, Chu XY, Yang B, Zhao M, Sun Y (2014). Downregulation of LKB1 suppresses stat3 activity to promote the proliferation of esophageal carcinoma cells. Mol Med Rep.

[17] Su Z, Yang Z, Xu Y, Chen Y, Yu Q (2015). Apoptosis, autophagy, necroptosis, and cancer metastasis. Mol Cancer.

[18] Chen Y, Liu Y, Zhou Y, You H (2019). Molecular mechanism of LKB1 in the invasion and metastasis of colorectal cancer. Oncol Rep.

[19] Li J, Liu J, Li P, Mao X, Li W, Yang J (2014). Loss of LKB1 disrupts breast epithelial cell polarity and promotes breast cancer metastasis and invasion. J Exp Clin Cancer Res.

[20] Zheng G, Song K, Zhao Y (2019). Liver kinase B1 suppresses the metastasis and angiogenesis of lung cancer: involvement of the Shh signaling pathway. Neoplasma.

[21] Zhang W, Ding Y, Zhang C, Lu Q, Liu Z, Coughlan K (2017). Deletion of endothelial cell-specific liver kinase B1 increases angiogenesis and tumor growth via vascular endothelial growth factor. Oncogene.

[22] Patel K, Foretz M, Marion A, Campbell DG, Gourlay R, Boudaba N (2014). The LKB1-salt-inducible kinase pathway functions as a key gluconeogenic suppressor in the liver. Nat Commun.

[23] Hollstein PE, Eichner LJ, Brun SN, Kamireddy A, Svensson RU, Vera LI (2019). The AMPK-related kinases SIK1 and SIK3 mediate key tumor-suppressive effects of LKB1 in NSCLC. Cancer Discov.

[24] Hong B, Zhang J, Yang W (2018). Activation of the LKB1-SIK1 signaling pathway inhibits the TGF-beta-mediated epithelial-mesenchymal transition and apoptosis resistance of ovarian carcinoma cells. Mol Med Rep.

[25] Yao YH, Cui Y, Qiu XN, Zhang LZ, Zhang W, Li H (2016). Attenuated LKB1-SIK1 signaling promotes epithelial-mesenchymal transition and radioresistance of non-small cell lung cancer cells. Chin J Cancer.

